# Well-being in high-risk pregnancy: an integrative review

**DOI:** 10.1186/s12884-020-03190-6

**Published:** 2020-09-11

**Authors:** Kobra Mirzakhani, Abbas Ebadi, Farhad Faridhosseini, Talaat Khadivzadeh

**Affiliations:** 1 Nursing and Midwifery Care Research Center, Mashhad University of Medical Sciences, Mashhad, Iran; 2 Department of Midwifery, School of Nursing and Midwifery, Mashhad University of Medical Sciences, Mashhad, Iran; 3 Behavioral Sciences Research Center, Life style institute, Baqiyatallah University of Medical Sciences, Tehran, Iran; 4 Nursing Faculty, Baqiyatallah University of Medical Sciences, Tehran, Iran; 5 Psychiatry and Behavioral Sciences Research Center, Mashhad University of Medical Sciences, Mashhad, Iran

**Keywords:** Well-being, Pregnancy, High-risk pregnancy

## Abstract

**Background:**

A prerequisite to the interventions for well-being improvement in high-risk pregnancy (HRP) is to make the concept clear, objective, and measurable. Despite the wealth of studies into the concept of well-being in HRP, there is no clear definition for it. This study aimed to explore the concept of well-being in HRP.

**Methods:**

This integrative review was conducted using the Whittemore and Knafl’s approach. A literature search was done without any data limitation in dictionaries, thesauruses, encyclopedias, well-being-related textbooks, midwifery, psychology, and mental health journals, and Iranian and international databases. The most primary inclusion criterion was relevance to well-being in HRP. The full-texts of all these articles were assessed using the checklists of the Joanna Briggs Institute. Data were analyzed through the constant comparison method and were managed using the MAXQDA 10 software. Meaning units were identified and coded. The codes were grouped into subcategories and categories according to the attributes, antecedents, and consequences of well-being in HRP.

**Results:**

Thirty articles were included in the review, from which 540 codes were extracted. The codes were grouped into seven main attributes, eight main antecedents, and five main consequences of well-being in HRP. The four unique dimensions of well-being in HRP are physical, mental-emotional, social, and spiritual well-being. These dimensions differentiate well-being in HRP from well-being in low-risk pregnancy and in non-pregnancy conditions.

**Conclusion:**

As a complex and multidimensional concept, well-being in HRP refers to the pregnant woman’s evaluation of her life during HRP. It includes physical, hedonic, and eudaimonic components. The assessment of well-being in HRP should include all these components.

## Background

Pregnancy is a critical period in women’s lives because pregnant women experience different physical, mental, and social changes. Ineffective coping with such changes cause them serious problems [1]. Although pregnancy is a physiologic phenomenon, some conditions may endanger maternal or fetal health and thereby, turn pregnancy into a high-risk pregnancy (HRP) and cause women to experience stressful conditions [2]. Almost 22% of pregnant women face with HRP [1].

HRP is associated with different physical complications which in turn can cause mood changes and mental and social problems. Studies showed that women with HRP experience negative feelings such as restlessness, fear, loss of control, disability, anger, and anxiety [3–5]. A qualitative study also showed that besides medical problems, women with HRP experience behavioral, affective, and emotional problems as well as problems in personal and familial role performance. Moreover, they are at risk for sociocultural and financial strains and uncontrollable feelings such as uncertainty, concern, and insecurity [4]. Consequently, HRP threatens women’s well-being [4, 6].

Well-being is a widely-used concept in different disciplines [7], particularly in health-related disciplines. Yet, there is no clear definition for it and controversies exist over its definition [8]. Oxford dictionary defines well-being as “the state of being comfortable, healthy, or happy” with the three dimensions of physical, emotional, and psychological well-being [9]. Similarly, Cambridge dictionary defines it as “the state of feeling healthy and happy” [10]. Mosby’s medical dictionary defines it as the “achievement of a good and satisfactory existence as defined by the individual”. In nursing, well-being is considered as personal satisfaction with health status as expressed by individuals [11]. The World Health Organization considers well-being as a keyword in the definition of health, with physical, spiritual, and social dimensions. It also uses well-being to define mental health as the following: “a state of well-being in which every individual realizes his or her own potential, can cope with the normal stress of life, can work productively and fruitfully, and is able to make a contribution to his or her community” [12]. Controversies also exist over the definition of well-being in HRP. Some studies vaguely addressed well-being in HRP [13] and some considered well-being in HRP as the lack of depression, anxiety, or other psychological disorders [14–17]. Another study also equated well-being with physical health [18].

Provision of a clear and objective definition for well-being is a prerequisite to its assessment and improvement among women with HRP [19]. Because of the lack of such definition, the present integrative review was conducted to explore the concept of well-being in HRP.

## Methods

This integrative review was conducted using the Whittemore and Knafl’s approach. Integrative review allows the assessment of both empirical and non-empirical studies. In this method, concepts are defined, theories and evidence are reviewed, and methodological issues related to the intended concept are analyzed to extract its attributes, antecedents, and consequences, further clarify the concept, and provide a better understanding and clearer definition for it [20, 21]. The Whittemore and Knafl’s integrative review approach consists of five stages, namely problem (or concept) identification, literature search, data evaluation, data analysis, and presentation.

### Stage 1. Problem identification

The first stage of each review is to clearly identify the intended problem or concept, acquire an in-depth understanding of it, determine the appropriate sampling framework, and clarify the relevant questions [21]. The main questions of the present study were: “How can the concept of well-being in HRP be clearly defined?” “How is the concept of well-being in HRP defined in different disciplines and communities?” and “What are the uses of the concept of well-being in HRP in the literature?”

### Stage 2. Literature search

**Fig. 1:**
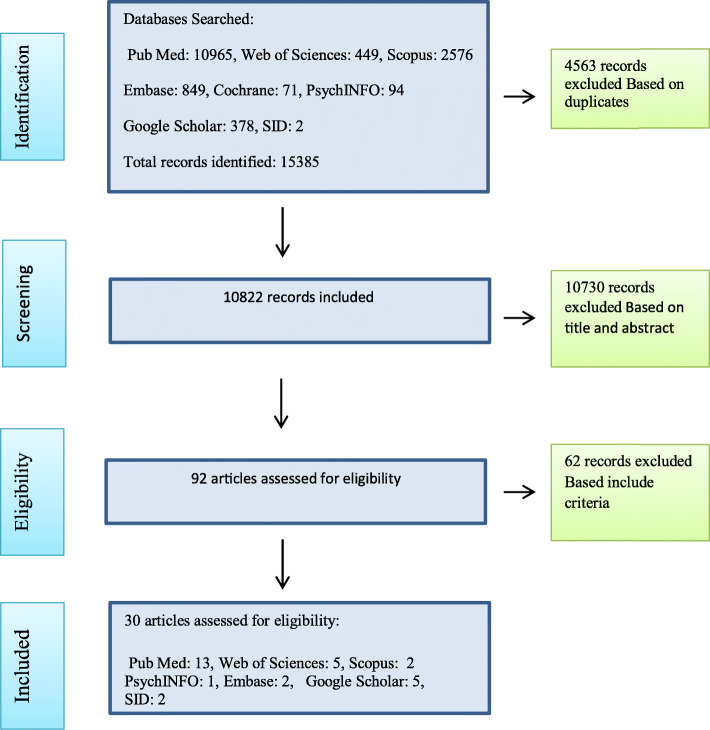
The PRISMA flow diagram

An online search was conducted in the following Iranian and international databases, SID, Irandoc, Magiran, Google Scholar, CINAHL, Medline via OVID, Embase, PsychINFO, NLM Gateway, Web of Science, Cochrane, ProQuest, ScienceDirect, and Scopus. Search keywords were “high-risk”, “complicated”, “pregnancy”, “maternal”, “mother”, “women”, “wellbeing”, and “well-being”. These keywords were combined using Boolean operators “AND” and “OR”. The search protocol was not limited to any time period. The reference lists of all retrieved articles were also assessed for any relevant study. Dictionaries, thesauruses, and encyclopedias were also searched to determine the definitions, synonyms, and expressions used to describe well-being in HRP. Manual search was also conducted in midwifery, psychology, and mental health journals as well as well-being-related textbooks. The literature search was done and reported using the four-step Preferred Reporting Items for Systematic Reviews and Meta-analysis (PRISMA) guideline (Fig. 1). The four steps of this guideline are record identification, record screening, eligibility assessment, and inclusion [22].

Records were included if they were related to well-being among women with HRP and had been published in English or Persian. Records addressed well-being in relation to healthy pregnancy, delivery, postpartum period, fetal death, and perineal injuries were not included. In order to reduce record selection bias, literature search and eligibility assessment were performed by two independent researchers (i.e. the first and the corresponding authors). They assessed the titles and the abstracts of the retrieved articles and excluded the ineligible articles.

The primary literature search yielded 15,385 records, 4563 of which were excluded due to duplication. The EndNote reference management software was used to determine and exclude the duplicated records. During the screening step, the titles and the abstracts of the remaining articles were assessed and 10,730 articles were excluded because they were not relevant to the study aim and questions. Moreover, 62 articles were excluded due to ineligibility. Finally, thirty articles were included in the study (Fig. 1) (Table 1). During literature search, we found no textbook relevant to the study aim and questions.

**Table 1:** Characteristics of Included Studies

No.	Authors/Year/Country	Aims	Study design	Well-being definition
1	Fellmeth/2018/Thai-Myanmar	To explore experiences of perinatal depression among refugee and labor migrant women living along the Thai-Myanmar border [23]	Qualitative	Well-being is defined by mental health and lack of prenatal depression.
2	Göbel/2018/Denmark	To systematically report and summarize the methodology and results of studies examining the relation between prenatal anxiety and maternal-fetal bonding [24]	Explanatory analysis and systematic review	Negative well-being includes maternal anxiety, distress, and depression.
3	Gentile/2017/Italy	To assess effects of intrauterine exposure to maternal depression or depressive symptoms in order to help clinicians to balance the risk of fetal complications with the effects of maternal mood disorders [25]	Systematic review	Negative well-being include depression and emotional distress.
4	Queyam/2017/Eastern Macedonia	To assess and compare different techniques to non-invasively measure physiological parameters for the purpose of monitoring fetomaternal well-being [26]	Review of methods	Maternal well-being is monitored through physiological parameters.
5	Fairbrother/2017/Canada	To assess the prevalence and incidence of anxiety disorders among pregnant women with varying levels of maternal, obstetric, and fetal risk in pregnancy [27]	Cohort	Well-being is implicitly defined as the absence of perinatal anxiety.
6	Nasiri-Kanari/2017/Iran	To examine the relationship of subjective well-being and happiness with pregnancy anxiety among pregnant women in Tabriz [28]	Descriptive correlational	Subjective well-being and happiness are two positive factors in decreasing pregnancy anxiety.
7	Linden/2016/Sweden	To explore well-being and diabetes management in women with type 1 diabetes mellitus in early pregnancy and To investigate associations among perceived well-being, diabetes management, and maternal characteristics [29]	Multi-centre randomized controlled trial	Well-being is defined by great self-efficacy for blood sugar control and low level of anxiety.
8	Saraian/2016/Iran	To compare perceived social support and psychological well-being between pregnant women with surrogacy, assisted reproductive technology (ART), and natural fertility [30]	Descriptive	Ryff’s definition of psychological well-being.
9	Taylor/2015/United Kingdom	To examine the case for universal thyroid screening in pregnancy and scrutinize this against established criteria for screening [31]	Review	Thyroid dysfunction denotes poor maternal well-being.
10	Roberts/2014/Australia	To explore pregnancy-related anticipated and experienced stress and promoting psychological well-being among women with phenylketonuria [32]	Qualitative	Well-being is implicitly defined by the absence of stress, concern, feeling of guilt, and physical problems and presence of positive social interactions.
11	Ngoma/2012/Japan	To explore support-seeking behavior among Japanese mothers at high risk for mental health problems [33]	Survey	Well-being in HRP is implicitly defined by mental health and lack of maternal depression.
12	McCarthy/2011/New Zealand, Australia, Ireland, and United Kingdom	To investigate the association between hyperemesis gravidarum and altered cognitive, behavioral and emotional well-being in pregnancy [14]	Prospective cohort	Well-being in HRP has cognitive, behavioral, and emotional dimensions and is implicitly defined by and lack of anxiety, stress, depression, and behavioral responses to pregnancy.
13	Bigelow/2011/USA	To assess bed rest versus normal activity for various complications of pregnancy [34]	Review	Well-being in HRP has physical, psychological, interpersonal, financial, spiritual and societal dimensions.
14	Woods/2010/USA	To identify factors associated with high antenatal psychosocial stress and describe the course of psychosocial stress during pregnancy [35]	Longitudinal study	Well-being in HRP is implicitly defined by lack of psychosocial stress.
15	Tough/2010/Canada	To identify maternal well-being and its association with the risk of developmental problems in children at school entry [36]	Cohort study	Well-being is defined as mental health.
16	Leeners/2008/German	To investigate the experience of women with hypertensive diseases in pregnancy [37]	Exploratory and descriptive	Well-being in HRP is implicitly defined by lack of psychosocial strain, stress, fear, uncertainty, dissatisfaction, and feeling of guilt.
17	Stark/2007/United States	To examine the relationship between maternal perceived stress and health-promoting self-care behaviors in women with HRP [38]	Descriptive-correlational	Well-being in HRP is implicitly defined by lack of stress, fear, and anxiety.
18	Dunn/2007/United States	To examine relationships among anxiety, depression, and spiritual well-being in three groups of women (non-pregnant, normal pregnancy, HRP on bed rest) [39]	Descriptive-correlational	“Spiritual well-being has two dimensions, namely existential and religious. Existential spiritual well-being refers to a sense of meaning and purpose in life. Religious spiritual well-being refers to having a focus on one’s relationship with God or a higher power”
19	Black/2007/United States	To investigate the relationships of psychological stress, preeclampsia/gestational hypertension symptoms, confidence in self-monitoring, well-being, and perceived social support with preeclampsia/gestational hypertension disease progression in outpatient women [40]	Retrospective correlational and comparative	Well-being is an abstract level of health. Well-being in HRP has two dimensions, namely physical (including fitness) and psychological (including mood, affect, and contentment).
20	Sayil/2007/Turkey	To examine demographic, environmental, and personality factors related to maternal well-being [41]	Cohort	Well-being in HRP is implicitly defined by lack of maternal anxiety and depression.
21	Breen/2006/Canada	To explores the connections between spirituality, health, and HRP [42]	Review	Well-being in HRP has three dimensions, namely physical, mental, and spiritual. Spiritual well-being affects physical and mental well-being.
22	Markovic/2006/Australia	To investigate how the Australian social context and the health care system intersect with and shape the experiences of individual women [43]	Grounded theory	Negative well-being in HRP is defined by lack of control over body and feelings of concern, uncertainty, and threat.
23	Giurgescu/2006/USA	To investigate whether prenatal coping strategies (preparation for motherhood, avoidance, positive interpretation of events, and prayer) mediate the effects of uncertainty and social support on the psychological well-being of women with HRP [44]..	Cross-sectional	Well-being is defined by lack of fear, anxiety, emotional distance from the baby, depression, loneliness, dysphoria, anxiety, hostility, fear, and loss of control.
24	Hobel/2003/USA	To assess the role of psychosocial and nutritional stress on poor pregnancy outcome [45]	Review	Well-being is implicitly defined by lack of psychosocial stress, fears, and anxiety.
25	Levy-Shiff/2002/Israel	To empirically explore psychosocial functioning in HRP and its relation to infant developmental outcomes by focusing on the pregnancies of women with presentational diabetes mellitus and women with gestational diabetes mellitus [46]	Cohort	Well-being is defined by lack of health-related stress. It consists of three components, namely physical exhaustion, emotional exhaustion, and psychological exhaustion.
26	Paarlberg/1996/Netherlands	To examine the psychosocial predictors of well-being and of pregnancy-related complaints throughout pregnancy [47]	Cohort	Well-being is defined as adequate physical and mental functioning.
27	Langer/1996/Latin American	To examine the impact of a psychosocial support program on women’s psychosocial conditions and on their role as mothers To explore the impact of a psychosocial support program on well-being and satisfaction with reproductive experience [48]	Randomized controlled trial	Well-being in HRP is implicitly defined by lack of psychosocial distress and maternal anxiety.
28	Oakley/1991/United Kingdom	To assess the views and experiences of high-risk mothers with respect to the use of medical care [49]	Randomized controlled trial	Well-being is defined as satisfaction and absence of stress.
29	Lang/1989/German	To describe the current situation of diabetic pregnancies in comparison to non-diabetic pregnancies in a Central European setting [50]	Survey	Well-being is implicitly defined by lack of maternal mortality and morbidity.
30	Cunningham/1979/Texas	To assess the prophylactic transfusions of normal red blood cells during pregnancies complicated by sickle cell hemoglobinopathies [51].	Cohort	Well-being is defined as successful control of physical stressors such as pain, edema, weight, blood pressure and laboratory parameters.

### Stage 3. Data evaluation

The 30 articles included in the integrative review were three clinical trials [48, 49, 52], eight descriptive studies [28, 30, 33, 37–40, 50], five reviews [26, 31, 34, 42, 45], two systematic reviews [24, 25], eight cohort studies [14, 27, 35, 36, 41, 46, 47, 51], three qualitative studies [23, 32, 43], and a cross-sectional study [44] (Table 1). The full-texts of all these articles were assessed using the checklists of the Joanna Briggs Institute (JBI). The JBI developed different design-specific checklists for the assessment of studies with different designs [53]. The first and the corresponding authors of the present study independently assessed the included studies using design-specific JBI checklists and resolved their disagreements through seeking other authors’ comments. Articles obtained at least 65% of the total score of the JBI checklists were included in the final analysis [54, 55]. The following questions were also used for eligibility assessment: “Does the article describe well-being in HRP?” “Does the article provide an explicit or implicit explanation or instances of well-being in HRP?” “Has the article introduced or assessed the attributes, antecedents, or consequences of the concept of well-being in HRP?”

### Stage 4. Data analysis

Data analysis was performed using the four-step constant comparison method. The four steps of this method are data reduction, data display, data comparison, and conclusion drawing and verification. Data reduction refers to the coding of the data in a manageable framework. Excerpts of the included articles which were relevant to the concept of well-being in HRP were considered as meaning units and were coded. In the second step, the codes were displayed in two tables (Tables 2 and 3). In the third step, the codes were frequently compared with each other, similar codes were grouped into subcategories, and similar subcategories were grouped into categories. In the fourth step, the results were arranged into the following main categories: definitions, attributes, antecedents, and consequences [21, 56]. The MAXQDA 10 software was employed for managing the data.

**Table 2:** The codes and subcategories of the controlled physical conditions main category of well-being in HRP

Reference	Codes	Subcategories	Category
Barwin et al. [45]	Maternal well-being assessment through assessing physiologic changes	Successful control of physiologic parameters	Controlled physical conditions
Queyam et al. [39]	Maternal and fetal well-being monitoring through assessing physiologic parameters		
Cunningham et al. [40]	Well-being as normal laboratory findings		
	Well-being as controlled blood pressure		
Levyshiff et al. [36]	Physical fatigue as a dimension of well-being and distress	Successful control of physical health conditions	
Black [47]	Fitness as a dimension of physical well-being		
Cunningham et al. [40]	Well-being as the absence of pain		
	Well-being as the absence of edema

**Table 3:** The categories and subcategories of the attributes, antecedents, and consequences of the concept of well-being in HRP

Subcategories	Categories	Components
Well-being as an abstract concept; Well-being as a multidimensional concept; Well-being and health as intertwined concepts	Well-being as a multidimensional and complex concept	The attributes of well-being in HRP
Successful control of physiologic parameters; Successful control of physical health conditions	Controlled physical conditions	
Anxiety; Depression; Stress; Satisfaction with the present conditions; Satisfaction with laboratory tests; No feeling of loneliness; Feeling guilty at adverse pregnancy outcomes; Feeling of vitality; and Feeling of hope	Controlled mood, emotions, and affections	
Fear over adverse pregnancy outcomes; Uncertainty over pregnancy outcomes; Concern over adverse pregnancy outcomes; and perceiving danger	Perceived threat	
Feeling of self-efficacy; Behavioral response to pregnancy; Damages to maternal roles	Self-efficacy and competence for multiple role performance	
Maintaining positive social interactions; Maintaining positive relationships with spouse and others	Maintained social relationships	
Spiritual components of well-being in HRP; Spiritual well-being and health as intertwined concepts	Meaning seeking and relationship with the Creator	
Personal characteristics; Social position; Financial security	Personal and socioeconomic characteristics	The antecedents of well-being in HRP
Physical suffering as a predictor of well-being; Tension due to pregnancy-related physical symptoms; Hospitalization-related functional limitation	Physical tensions	
Access to health services; Easy intake of health services; Appropriateness of health services; Free and informed choice of health services	Availability and perceived quality of health services	
History of psychological disorders; History of negative life events; Personal competence; Pregnancy wantedness	Psychological context	
Mental, emotional, and legal support; Informational support; Relationship with a successful peer model	Social support	
Friendly relationships with spouse and others; Empathetic interactions with spouse; Empathetic interactions with nurses and midwives	Interpersonal relationships	
Coping strategies to have good feelings; Health-promoting behaviors	Coping strategies	
Spiritual beliefs; Engagement in religious rituals	Spirituality and religiosity	
Well-being as a facilitator to the achievement of physical health; Mood improvement; Anxiety reduction; Mental health improvement	Maternal health	The consequences of well-being in HRP
Poor maternal well-being as a factor which negatively affects maternal image of the fetus as a real person; Mother-fetus emotional belongingness	Mother-fetus emotional attachment	
Adverse pregnancy outcome in case of poor well-being; Premature delivery in case of poor well-being	*Success in pregnancy*	
Fetus’s physical and behavioral responses; Fetus’s hormonal, nervous, and epigenetic changes; Fetal well-being as a reflection of maternal well-being	Fetal well-being	
Neonatal physical outcomes; Hormonal and nervous changes during infancy and childhood; Neuromotor outcomes during infancy and childhood; Behavioral outcomes during infancy, childhood, and adolescence; Changes in growth and development	Outcomes related to child’s future	

## Results

During the integrative review of the 30 articles, 540 codes about well-being in HRP were extracted (Table 2). Findings revealed that there were different definitions for well-being in HRP, none of which was comprehensive. Thus, the attributes, antecedents, and consequences of the concept were extracted (Table 3) in order to provide a better understanding about it.

### The attributes of well-being in HRP

Through the analysis of the explicit and the implicit definitions of well-being in HRP, seven attributes, symptoms, or components were identified for the concept. These attributes were as the following: well-being as a multidimensional and complex concept [29, 52], controlled physical conditions [26, 32], controlled mood, emotions, and affections [14, 29], perceived threat [32, 48], self-efficacy and competence for multiple role performance [29, 44], maintained social relationships [23, 32], and meaning seeking and relationship with the Creator [39].

#### Well-being as a multidimensional and complex concept

Well-being in HRP is a multidimensional and complex subjective concept which may change during pregnancy. It is integrated with the concept of health and is considered as the abstract understanding of health [29, 52].

#### Controlled physical conditions

Well-being in high risk pregnancy refers to the successful control of physiologic parameters [26, 57] and successful control of physical health conditions [32, 51]. In other words, it denotes that the physical problems of HRP have been controlled and are bearable [47, 51].

#### Controlled mood, emotions, and affections

The components of negative well-being in HRP are anxiety [14, 27, 28, 41, 48], depression [14, 24, 44, 47], and stress [14, 24, 32, 40, 44, 57]. A woman with HRP may feel well-being when she is satisfied with her conditions, her positive feelings overweigh her negative feelings [32, 37, 47], and does not experience unpleasant affective and emotional feelings [23, 44, 46]. Thereby, well-being in HRP is achieved when mood, emotions, and affections are under control.

#### Perceived threat

In HRP, women with poor well-being feel concern and have fear over the adverse consequences of pregnancy [37, 44, 45]. They may feel that they and their fetuses are at risk for threats and hence, are uncertain about pregnancy outcomes [37, 40, 41, 43, 44].

#### Self-efficacy and competence for multiple role performance

One of the key components of well-being in HRP is self-efficacy and self-control for showing appropriate responses to pregnancy [44, 52]. Limitation of behaviors and activities [14, 38], a sense of strain, and damages to maternal roles in family [23, 32] are indicative of poor well-being in HRP.

#### Maintained social relationships

Damage to social activities and relationships and disturbances in marital relationships also indicate poor well-being in HRP [32].

#### Meaning seeking and relationship with the Creator

Well-being in HRP is an abstract concept with two existential and religious dimensions. It is closely related to pregnant women’s mental and general health [34, 39, 42].

### The antecedents of well-being in HRP

Data analysis revealed that the antecedents of well-being in HRP were personal and socioeconomic characteristics, physical tensions, availability and perceived quality of health services, psychological context, social support, interpersonal relationships, coping strategies, and spirituality and religiosity. These are explained in the following.

#### Personal and socioeconomic characteristics

Personal and socioeconomic characteristics such as age, number of pregnancies, educational level, marital status, income level, and employment status are related to well-being in HRP [33, 41, 44, 47, 48]. Accordingly, women who are nulliparous, younger [33], with a lower educational level [44], lower financial status, and financial insecurity are at greater risk for poorer well-being [23, 41, 47, 48].

#### Physical tensions

Physical problems such as pain, nausea, and vomiting are the predictors of poor well-being in HRP. The tension caused by the physical problems of pregnancy, development of pregnancy to an HRP, and hospitalization-induced functional limitations can be associated with psychological distress and poorer well-being in HRP [14, 23, 24, 31–33, 35, 37, 39, 40, 44, 47, 49, 57].

#### Availability and perceived quality of health services

Good access to healthcare services gives women with HRP the sense of well-being [32, 37, 49]. Contrarily, limited access to diagnostic, therapeutic, and intensive care services are associated with fear, stress, and poorer well-being [32, 49]. Moreover, mothers who perceive that health services are appropriate and are not disturbing feel higher levels of well-being [32, 38]. Informed and free choice of health services also improves satisfaction and well-being among pregnant women [43, 49].

#### Psychological context

The history of psychological disorders, critical negative life events [23, 35, 37, 47], adverse pregnancy outcomes in previous pregnancies [37, 44, 49], and infertility is associated with lower levels of well-being in HRP [30, 49]. Conversely, wanted pregnancy and personal competencies such as good self-esteem improve well-being in HRP [29, 33, 41, 47, 48].

#### Social support

Social and emotional support received from family, spouse, peers, neighbors, colleagues, and healthcare providers can improve well-being in pregnancy [23, 32, 37, 40, 41, 44, 47, 48]. Moreover, supportive rules, regulations, and services at workplace and in communities for women with HRP are associated with higher levels of well-being [41]. Informational support and quality educational services by healthcare providers and the possibility of communication with successful peer models can also improve well-being among women with HRP [37, 38, 43, 49].

#### Interpersonal relationships

Healthy and committed marital relationships improve well-being among pregnant women, while poor marital relationships reduce it [23, 48]. Moreover, healthcare providers’ empathy with pregnant women improves their well-being [37, 49].

#### Coping strategies

Coping strategies can also affect well-being through reducing stress and depression, increasing engagement in recreational activities [23, 32, 37, 44], and promoting adherence to health-promoting behaviors [35, 38, 48].

#### Spirituality and religiosity

Spiritual and religious beliefs and engagement in religious rituals can also improve well-being among women with HRP [39].

### The consequences of well-being in HRP

The integrative review of the existing literature revealed five main consequences for well-being in HRP. These consequences were maternal health [31, 37], mother-fetus emotional attachment [24, 33], success in pregnancy [14, 45, 49], fetal well-being [24, 25, 36, 45], and consequences related to child’s future [25, 36, 50].

#### Maternal health

Pregnant women with lower well-being are less likely to engage in health-promoting behaviors [33]. Well-being is a facilitator to the achievement of physical health and is associated with lower morbidity and mortality rates [14, 29]. Well-being also improves mental health among pregnant women [39].

#### Mother-fetus emotional attachment

Poor maternal well-being ruins maternal image of the fetus as a real person [24] and thereby, negatively affects mother-fetus emotional attachment [33].

#### Success in pregnancy

Well-being in pregnancy is associated with a successful pregnancy [14, 45]. Contrarily, poor well-being in pregnancy is associated with adverse pregnancy outcomes [45, 49]. For example, it can increase the likelihood of preterm delivery .

#### Fetal well-being

Fetal well-being directly depends on maternal well-being, so that changes in maternal well-being are associated with changes in fetal well-being [58, 59]. For example, maternal depression or emotional distress during pregnancy can result in retarded fetal growth [45], fetal hyperactivity, and irregular fetal heart rhythm. Hormonal, nervous, and epigenetic changes in the fetus are also observed in case of poor maternal mental-emotional well-being [25]. Moreover, poor maternal physical well-being can be associated with low birth weight and preterm delivery. Contrarily, improvement in maternal wellbeing reduces adverse fetal outcomes [37].

#### Consequences related to child’s future

Poor maternal well-being can result in adverse neonatal and infantile consequences such as low birth weight, prematurity, increased neonatal mortality rate, hormonal and nervous changes, and motor and behavioral disorders. Moreover, the child of mothers with poor well-being in pregnancy may develop growth and development disorders, autism spectrum disorders, and behavioral and criminal disorders later in adolescence [24, 25, 36].

### The definition of well-being in HRP

Well-being in HRP is a multidimensional, complex and abstract subjective concept and a cognitive and emotional self-evaluation of one’s own life in HRP. Its four main dimensions are physical, mental-emotional, social, and spiritual well-being. These dimensions are interrelated and affect each other. In the physical dimension, pregnant women with good well-being have control over their physiologic parameters and physical health conditions. In the mental-emotional dimension, women with good well-being have more positive feelings towards their pregnancy, so that they have lower concerns, fears, depression, anxiety, and stress in relation to the adverse maternal and fetal outcomes of pregnancy, are more satisfied with their conditions, and feel greater self-efficacy for managing their conditions in HRP. In the social dimension, women with good well-being in HRP are able to fulfill their roles and maintain their social interactions and have positive interpersonal relationships. In the spiritual dimension, they have meaning and purpose in life and pregnancy and establish relationships with God or a supreme power. Well-being in HRP is affected by personal and socioeconomic characteristics, physical, mental, social, environmental, and behavioral contexts, and spiritual and religious beliefs. HRP among women with good well-being is associated with lower adverse maternal, fetal and infantile consequences.

## Discussion

This integrative review aimed to explore the concept of well-being in HRP and identify its attributes, antecedents, and consequences. Findings indicated that multidimensionality, complexity, and subjectivity were among the characteristics of well-being in HRP. These characteristics are also for the concepts of general well-being [60–62] as well as situation-specific well-being such as perinatal well-being and well-being in other disciplines [63, 64]. Yet, complexity, multidimensionality, and subjectivity are not unique to the concept of well-being; rather, many other concepts such as quality of life and comfort are multidimensional, complex, and subjective. Despite their similar characteristics, concepts such as quality of life and comfort cannot interchangeably be used with the concept of well-being [65, 66].

Findings also showed that the four dimensions of well-being in HRP concept were physical, mental-emotional, social, and spiritual well-being. Similarly, a former systematic review reported that child well-being includes the five physical, psychological, cognitive, social, and economic dimensions which vary from negative to positive well-being [67]. Well-being in pregnancy may also vary from positive to negative extremes [63]. Compared with low-risk pregnancy, negative well-being is perceived more commonly in HRP [68]. Although the dimensions of well-being in HRP are in some ways similar to the dimensions of well-being in other conditions, their definitions and subcategories are different. Well-being refers to self-perception and self-evaluation of one’s own position in life with respect to goals, expectations, standards, and concerns. Consequently, well-being in HRP differs from HRP in low-risk pregnancy and non-pregnancy conditions, though well-being is a complex and multidimensional concept in all conditions. Some studies suggested that pregnancy is associated with feelings of happiness, satisfaction, and self-worth because reproductive role is socially well-accepted. A concept analysis of the perinatal well-being concept defined well-being as the level of adaptation to pregnancy-induced physical and emotional changes which has physical, psychological, social, spiritual, economical, and ecological dimensions and is affected by cultural, social, spiritual, ecological, and economic factors [63]. In the present study, economic and ecological factors were determined as the antecedents of the well-being concept not as its attributes. Another study defined well-being in low-risk pregnancy as the absence of any illness [69]. However, the present study showed that well-being can also be defined in HRP, where pregnant women suffer from a disorder of illness.

In the presents study, the physical well-being dimension was one of the most common attributes of well-being in HRP. Physical well-being is necessary and important to achieve general well-being and health [66, 70]. This dimension refers to the successful control of physiologic parameters and physical health conditions [26, 32, 43, 51]. Physical disorders can turn normal pregnancy into HRP [2, 71]. Therefore, physical disorders, perceived and expressed as ailment, are the first problems experienced by women with HRP compared with normal pregnancy. In other words, a woman feels acceptable level of well-being when her physical problems are under control, her physiologic parameters are in safe ranges [72], and she is able to cope with physical stress [51]. Physical well-being also refers to functional ability and pertains to environmental factors such as facilities, equipment, and habitation standards [60, 73]. A previous study into the well-being of doctorate students also found physical well-being, health maintenance, and work-life balance as the components of well-being [64]. However, a study into the concepts of well-being and quality of life reported that the core of well-being is its psycho-spiritual dimensions, happiness, and inner energy and did not consider physical well-being as a key component of well-being [65]. Some scholars believe that the physical dimension of well-being is more evident in medicine and nursing [65, 71]. Physical tensions and suffering [14, 32, 47] and perceived quality of health services were among the antecedents of physical well-being in HRP in the present study. Thus, reducing pregnant women’s physical tensions and suffering and improving their access to quality health services can improve their sense of well-being [32, 38, 43, 49].

Mental-emotional well-being was another dimension of well-being in HRP. The characteristics of mental-emotional well-being are successful control over mood, emotions, and affections, perceived threat, sense of self-efficacy, and competence for multiple role performance. A pregnant woman may have low levels of well-being if she is experiencing depression, anxiety, and stress [14, 41, 47, 48, 74] and feels guilty and alone [19, 37, 46]. Contrarily, a woman with feelings of satisfaction [32, 37, 47–49], vitality [44], hopefulness [46], and successful control over mood and emotions will feel acceptable level of well-being in HRP.

Perceived threat is a component of psychological well-being. A pregnant woman with HRP may feel threat due to her fear and concern over the adverse outcomes of pregnancy and uncertainty about pregnancy outcomes. In other words, well-being in HRP largely depends on feelings of safety and security [40, 41, 43]. Safety is also a key component of well-being in other disciplines [64, 75, 76]. However, the difference between well-being in HRP and well-being in other disciplines pertains to pregnant woman’s confidence in her health, her fetus’s health, and pregnancy outcomes.

Perceived threat and successful control of mood, emotions, and affections in the psychological dimension of well-being in HRP refer to the hedonic approach to well-being. In this approach, well-being is defined based on pleasure, happiness, positive emotions [61, 62, 77], balance between positive and negative emotions, and happiness [78–80]. In other words, the components of psychological well-being are positive emotions and affections, life satisfaction, marital satisfaction, positive mood, and absence of depression and anxiety [81]. Accordingly, a person with great life satisfaction, deep feeling of pleasure, and low levels of negative emotions (such as discomfort, tension and anger) will have high levels of psychological well-being and vice versa [82, 83].

Findings revealed that the psychological context of a woman with HRP affects her well-being and thereby, it is an antecedent of her mental-emotional well-being. Studies reported that the history of psychological disorders, negative life events [23, 35, 37, 47], negative pregnancy outcomes [37, 44, 49], and infertility are associated with low levels of well-being among women with HRP [30]. On the other hand, effective coping strategies can reduce stress and improve well-being [23, 32, 37, 38, 84–88]. Therefore, interventions are needed to improve coping skills among women with HRP.

Self-efficacy for the management of HRP was another aspect of mental well-being in HRP in the present study. Most health and well-being theories assert that feeling control over thoughts, feelings, behaviors, environment [89, 90], body and high-risk conditions of pregnancy and personal ability to manage pregnancy and its complications are among the key components of well-being [29, 44].

We also found competence for multiple role performance as another aspect of psychological well-being in HRP. HRP may cause disturbances in personal and familial life, inefficiencies in role performance and thereby, give women feeling of incompetence in role performance [4]. Competence was also reported to be a component of well-being in other disciplines. For instance, a previous study assessed faculty well-being based on the Self-Determination Theory. The two main components of this theory are competence and autonomy [91]. Another study used a competence scale to assess well-being among the parents of preschool children with autism [92].

Maintaining marital, interpersonal, and social relationships was identified as the social dimension of well-being. In HRP, women experience problems in their personal, familial, and social life [4, 5] and hence, their well-being is damaged. Contrarily, maintaining normal social relationships and interactions can help women with HRP protect their well-being. We also found social support and interpersonal relationships as the antecedents of social well-being in HRP. Three earlier qualitative studies reported social support and empathetic relationships with spouse, healthcare providers, and society as the fundamental needs of women with HRP [5, 93, 94]. Social well-being is also among the key components of well-being in other disciplines [65, 67, 76]. For instance, active engagement in social activities, quality social relationships, and maintenance of positive relationships with spouse, peers, and relatives were reported as the components of general, geriatric, and perinatal well-being [60, 63, 72, 76]. A former concept analysis study also found dynamicity in interactions, maintenance of relationships, negotiation for growth and development, and social support as the attributes of the concept of well-being in older adults [95].

Meaning seeking and relationship with the Creator were extracted as the spiritual dimension of well-being in HRP. Spiritual well-being was conceptualized by Ellison in 1983 [96]. It has two dimensions, namely existential and religious well-being. Existence is the horizontal aspect of spiritual well-being and encompasses meaning and purpose in life, while religion is the vertical aspect of spiritual well-being and refers to relationship with God or a supreme power. Spiritual well-being has close relationship with physical and mental health [97]. Spiritual well-being helps women cope with HRP-associated stress [98]. It is affected by spirituality and engagement in religious rituals which were extracted in the present study as its antecedents.

Self-efficacy for managing HRP, competence for multiple role performance, maintained social relationships, positive interpersonal relationships, and meaning seeking and relationship with the Creator, which were identified as the components of well-being in the present study, are close to the underpinnings of the eudemonic approach to well-being. Ryff, a eudemonic theorist, holds that well-being should not simply be equated with greater pleasure; rather, it encompasses attempts for perfection and actualization of individual potentials. To define well-being, she combined theories in the following six areas: self-acceptance, positive relations with others, autonomy, environmental mastery, purpose in life, and personal growth. According to the eudaimonic approach, well-being is the ability to address three main psychological needs, namely autonomy, competence, and relationship [99–101]. A study, with the assumption that Japanese people have lower level of well-being compared with western people, reported that Japanese people’s well-being is closer to the eudaimonic approach than the hedonic approach [102]. Contrarily, people in the Latin American countries, who have lower socioeconomic status, have higher levels of hedonic well-being and hence, development in these countries necessitates interventions to improve eudaimonic well-being [76]. These findings denote that well-being assessment and improvement in HRP should be performed using a holistic approach to address not only positive feelings, pleasure, peace, and satisfaction, but also awareness of values and meaning in life [103].

The main outcomes of well-being are general health and quality of life [75, 76]. As pregnancy directly involves two persons, i.e. a pregnant woman and her fetus, the outcomes of well-being in pregnancy can affect the health of both woman and fetus. Maternal well-being can affect fetal health and well-being through affecting mother-fetus relationships and causing hormonal and behavioral changes in the fetus. Studies showed that depression and distress are among the components of poor well-being in HRP [14, 24, 32, 44, 57]. These components can affect the health and the future of the fetus. Maternal depression can also cause neuromotor impairments, growth and development disorders, speech disorders, and autism spectrum disorders during childhood and criminal behaviors during adolescence [25].

## Strengths and limitations

The strengths of the present study were its integrative review design and inclusion of studies without any date limitation. The limitations of the study were the inclusion of only English or Persian articles, inaccessibility of gray literature, and analyzing the data and interpreting the findings using a subjective approach.

## Conclusion

Well-being in HRP refers to the pregnant woman’s evaluation of her life during pregnancy. As a complex and multidimensional concept, well-being in HRP includes a physical component, hedonic components such as balance between positive and negative feelings, and three eudaimonic components, namely autonomy, competence, and relationship. Thus, well-being assessment and improvement programs need to address all these components. Due to the significant effects of well-being on both maternal and fetal health, policies and interventions are needed to improve the different aspects of well-bring in HRP. As previous studies used different instruments for well-being measurement, an integrative review is recommended to review all these instruments. Moreover, a comprehensive instrument should be developed for well-being measurement in HRP. That instrument should be tested in different cultures and settings.

This integrative review provides a clear understanding about well-being in HRP and hence, its results can be used in both theory and practice. Healthcare providers usually focus mainly on the physical aspect of health during HRP management and may neglect pregnant women’s perceptions, feelings, and experiences. Meanwhile, evidence shows that a very basic step in care delivery to women with HRP in countries with effective HRP management and low maternal mortality rate is the use of holistic woman-centered approaches. Therefore, healthcare providers need to not only manage HRP-associated physical health problems, but also pay careful attention to pregnant women’s feelings, satisfaction, and well-being.

## Data Availability

The data collected and analyzed in the present study are available through requesting the corresponding author.

## Abbreviation

HRP: High-risk pregnancy

## References

[1] James DK, Steer PJ, Weiner CP, Gonik B, Robson SC (2017). High-risk pregnancy: management options: Cambridge University press.

[2] Medeiros AL, Santos SR, Cabral RWL, Silva JPG, Nascimento NM (2016). Assessing nursing diagnoses and interventions in labour and high-risk pregnancies. Rev Gaucha Enferm.

[3] Rodrigues PB, Zambaldi CF, Cantilino A, Sougey EB (2016). Special features of high-risk pregnancies as factors in development of mental distress: a review. Trends Psychiatry Psychother.

[4] Oliveira DC, Mandú ENT (2015). Women with high-risk pregnancy: experiences and perceptions of needs and care. Escola Anna Nery.

[5] Kent RA, Yazbek M, Heyns T, Coetzee I (2015). The support needs of high-risk antenatal patients in prolonged hospitalisation. Midwifery..

[6] Abdollahpour S, Heydari A, Ebrahimipour H, Faridhosseini F, Khadivzadeh T (2019). The needs of women who have experienced “maternal near miss”: a systematic review of literature. Iran J Nurs Midwifery Res.

[7] Acton GJ (1994). Well-being as a concept for theory, practice, and research. Worldviews Evid-Based Nurs.

[8] Dodge R, Daly AP, Huyton J, Sanders LD (2012). The challenge of defining wellbeing. Int J Wellbeing.

[9] Dictionary OE (2008). Oxford english dictionary. Retrieved May.

[10] Dictionary C (2015). Cambridge dictionaries online.

[11] Mosby (2016). Mosby’s medical dictionary: Elsevier Health Sciences.

[12] WMhasow-b (2018). mheaM. Mental health: a state of well-being [cited Mental health: a state of well-being]. Mental health: a state of well-being.

[13] Allan W, Haddow J, Palomaki G, Williams J, Mitchell M, Hermos R (2000). Maternal thyroid deficiency and pregnancy complications: implications for population screening. J Med Screen.

[14] McCarthy FP, Khashan AS, North RA, Moss-Morris R, Baker PN, Dekker G (2011). A prospective cohort study investigating associations between hyperemesis gravidarum and cognitive, behavioural and emotional well-being in pregnancy. PLoS One.

[15] Cohen LS, Altshuler LL, Harlow BL, Nonacs R, Newport DJ, Viguera AC (2006). Relapse of major depression during pregnancy in women who maintain or discontinue antidepressant treatment. Jama..

[16] Yonkers KA, Wisner KL, Stewart DE, Oberlander TF, Dell DL, Stotland N (2009). The management of depression during pregnancy: a report from the American Psychiatric Association and the American College of Obstetricians and Gynecologists. Gen Hosp Psychiatry.

[17] Nguyen PH, DiGirolamo AM, Gonzalez-Casanova I, Pham H, Hao W, Nguyen H (2017). Impact of preconceptional micronutrient supplementation on maternal mental health during pregnancy and postpartum: results from a randomized controlled trial in Vietnam. BMC Womens Health.

[18] Nurzaireena Z, Azalea K, Azirawaty T, Jameela S, Muralitharan G (2017). Sickle cell disease: review of managements in pregnancy and the outcome in Ampang hospital, Selangor. World Acad Sci Eng Technol Int J Med Health Biomed Bioeng Pharm Eng.

[19] Cummins RA, Eckersley R, Pallant J, Van Vugt J, Misajon R (2003). Developing a national index of subjective wellbeing: the Australian Unity wellbeing index. Soc Indic Res.

[20] Broome ME (2000). Integrative literature reviews for the development of concepts. Concept development in nursing: foundations, techniques and applications.

[21] Whittemore R, Knafl K (2005). The integrative review: updated methodology. J Adv Nurs.

[22] Liberati A, Altman DG, Tetzlaff J, Mulrow C, Gøtzsche PC, Ioannidis JP (2009). The PRISMA statement for reporting systematic reviews and meta-analyses of studies that evaluate health care interventions: explanation and elaboration. PLoS Med.

[23] Fellmeth G, Plugge EH, Nosten S, Oo MM, Fazel M, Charunwatthana P (2018). Living with severe perinatal depression: a qualitative study of the experiences of labour migrant and refugee women on the Thai-Myanmar border. BMC Psychiatry.

[24] Goebel A, Stuhrmann LY, Harder S, Schulte-Markwort M, Mudra S (2018). The association between maternal-fetal bonding and prenatal anxiety: An explanatory analysis and systematic review. J Affect Disord.

[25] Gentile S (2017). Untreated depression during pregnancy: Short-and long-term effects in offspring. A systematic review. Neuroscience..

[26] Queyam AB, Pahuja SK, Singh D (2017). Non-Invasive Feto-Maternal Well-Being Monitoring: A Review of Methods. J Eng Sci Technol Rev.

[27] Fairbrother N, Young AH, Zhang A, Janssen P, Antony MM (2017). The prevalence and incidence of perinatal anxiety disorders among women experiencing a medically complicated pregnancy. Arch Women's Mental Health..

[28] Nasiri-Kanari F, Alivandi-Vafa M (2017). The Prediction of Pregnancy Anxiety on the Basis of Subjective Well-Being and Happiness of Pregnant Women in Tabriz. Depict Health..

[29] Linden K. Women with type 1 diabetes during pregnancy and postpartum Well-being and diabetes management. https://gupea.ub.gu.se/handle/2077/54536. Accessed 19 Feb 2018.

[30] Saraian E, Sajjadian I (2016). Comparison of Perceived Social Support and Psychological Well-being between Pregnant Women with Surrogacy, Assisted Reproductive Technology (ART) and Natural Fertility. J Nurs Educ.

[31] Taylor PN, Okosieme OE, Premawardhana L, Lazarus JH (2015). Should all women be screened for thyroid dysfunction in pregnancy?. Women’s Health.

[32] Roberts RM, Muller T, Sweeney A, Bratkovic D, Gannoni A (2014). Promoting psychological well-being in women with phenylketonuria: pregnancy-related stresses, coping strategies and supports. Mol Genet Metabol Rep.

[33] Ngoma AM, Goto A, Suzuki Y, Tsutomi H, Yasumura S (2012). Support-seeking behavior among Japanese mothers at high-risk of mental health problems: a community-based study at a city health center. Fukushima J Med Sci.

[34] Bigelow C, Stone J (2011). Bed rest in pregnancy. Mt Sinai J Med.

[35] Woods SM, Melville JL, Guo Y, Fan M-Y, Gavin A (2010). Psychosocial stress during pregnancy. Am J Obstetr Gynecol.

[36] Tough SC, Siever JE, Benzies K, Leew S, Johnston DW (2010). Maternal well-being and its association to risk of developmental problems in children at school entry. BMC Pediatr.

[37] Leeners B, Stiller R, Neumaier-Wagner P, Kuse S, Schmitt A, Rath W (2008). Psychosocial distress associated with treatment of hypertensive diseases in pregnancy. Psychosomatics..

[38] Stark MA, Brinkley RL (2007). The relationship between perceived stress and health-promoting behaviors in high-risk pregnancy. J Perinat Neonatal Nurs.

[39] Dunn LL, Shelton MM (2007). Spiritual well-being, anxiety, and depression in antepartal women on bedrest. Issues Ment Health Nurs.

[40] Black KD (2007). Stress, symptoms, self-monitoring confidence, well-being, and social support in the progression of preeclampsia/gestational hypertension. J Obstet Gynecol Neonatal Nurs.

[41] Sayil M, Güre A, Uçanok Z (2007). First time mothers’ anxiety and depressive symptoms across the transition to motherhood: associations with maternal and environmental characteristics. Women Health..

[42] Breen GV, Price S, Spirituality LM, pregnancy h-r (2006). Another aspect of patient care. AWHONN Lifelines.

[43] Markovic M, Manderson L, Schaper H, Brennecke S (2006). Maternal identity change as a consequence of antenatal hospitalization. Health Care Women Int.

[44] Giurgescu C, Penckofer S, Maurer MC, Bryant FB (2006). Impact of uncertainty, social support, and prenatal coping on the psychological well-being of high-risk pregnant women. Nurs Res.

[45] Hobel C, Culhane J (2003). Role of psychosocial and nutritional stress on poor pregnancy outcome. J Nutr.

[46] Levy-Shiff R, Lerman M, Har-Even D, Hod M (2002). Maternal adjustment and infant outcome in medically defined high-risk pregnancy. Dev Psychol.

[47] Paarlberg K, Vingerhoets A, Passchier J, Heinen A, Dekker G, Van Geijn H (1996). Psychosocial factors as predictors of maternal well-being and pregnancy-related complaints. J Psychosom Obstet Gynecol.

[48] Langer A, Farnot U, Garcia C, Barros F, Victora C, Belizan JM (1996). The Latin American trial of psychosocial support during pregnancy: Effects on mother’s wellbeing and satisfaction. Soc Sci Med.

[49] Oakley A (1991). Using medical care: the views and experiences of high-risk mothers. Health Serv Res.

[50] Lang U, Künzel W (1989). Diabetes mellitus in pregnancy. Management and outcome of diabetic pregnancies in the state of Hesse, FRG; a five-year-survey. Eur J Obstet Gynecol Reprod Biol.

[51] Cunningham FG, Pritchard JA, Mason R, Chase G (1979). Prophylactic transfusions of normal red blood cells during pregnancies complicated by sickle cell hemoglobinopathies. Am J Obstet Gynecol.

[52] Linden K (2018). Women with type 1 diabetes during pregnancy and postpartum Well-being and diabetes management.

[53] https://wiki.joannabriggs.org. Accessed 15 Sept 2017.

[54] Pati D, Lorusso LN (2018). How to write a systematic review of the literature. Health Environ Res Design J.

[55] Wright RW, Brand RA, Dunn W, Spindler KP (2007). How to write a systematic review. Clin Orthop Related Res (1976–2007).

[56] Walker LO, Avant KC (2005). Strategies for theory construction in nursing. 6rd ed. Pearson.

[57] Barwin BN, Dempsey A, Hurteau GD (1976). Graphic monitoring of labour. Canadian Med Assoc J.

[58] Majella MG, Sarveswaran G, Yuvaraj Krishnamoorthy KS, Arikrishnan K, Kumar SG. A longitudinal study on high risk pregnancy and its outcome among antenatal women attending rural primary health centre in Puducherry, South India. J Educ Health Promot. 2019. 10.4103/jehp.jehp_144_18. 10.4103/jehp.jehp_144_18 PMC6378831 30815483

[59] Heyden E, Wimalawansa S (2018). Vitamin D: Effects on human reproduction, pregnancy, and fetal well-being. J Steroid Biochem Mol Biol.

[60] Kiefer RA (2008). An integrative review of the concept of well-being. Holistic Nurs Pract.

[61] Diener E (2000). Subjective well-being: The science of happiness and a proposal for a national index. Am Psychol.

[62] Diener E (1984). Subjective well-being. Psychol Bulletin.

[63] Allan C, Carrick-Sen D, Martin CR (2013). What is perinatal well-being? A concept analysis and review of the literature. J Reprod Infant Psychol.

[64] Schmidt M, Hansson E (2018). Doctoral students’ well-being: a literature review. Int J Qual Stud Health Well-being..

[65] Pinto S, Fumincelli L, Mazzo A, Caldeira S, Martins JC (2017). Comfort, well-being and quality of life: Discussion of the differences and similarities among the concepts. Porto Biomed J.

[66] Prick BW, Steegers EA, Jansen AG, Hop WC, Essink-Bot M-L, Peters NC (2010). Well being of obstetric patients on minimal blood transfusions (WOMB trial). BMC Pregnancy Childbirth..

[67] Pollard EL, Lee PD (2003). Child well-being: A systematic review of the literature. Soc Indicators Res.

[68] Hatmaker DD, Kemp VH (1998). Perception of threat and subjective well-being in low-risk and high-risk pregnant women. J Perinatal Neonatal Nurs.

[69] Morrell C, Cantrell A, Evans K, Carrick-Sen D (2013). A review of instruments to measure health-related quality of life and well-being among pregnant women. J Reprod Infant Psychol.

[70] Csikszentmihalyi M, Csikszentmihalyi M (2014). Flow and the foundations of positive psychology. Toward a psychology of optimal experience.

[71] Van Otterloo LR, Connelly CD (2016). Maternal risk during pregnancy: a concept analysis. J Clin Nurs.

[72] Butalia S, Audibert F, Côté A-M, Firoz T, Logan AG, Magee LA (2018). Hypertension Canada’s 2018 guidelines for the management of hypertension in pregnancy. Can J Cardiol.

[73] Jahani Shourab N, Ghaffari Sardasht F, Jafarnejad F, Esmaili H (2013). Assessment of prenatal care process based on donabedian model in Mashhad health centers. Iranian J Obstet Gynecol Infertil.

[74] Gilbert L, Gross J, Lanzi S, Quansah DY, Puder J, Horsch A (2019). How diet, physical activity and psychosocial well-being interact in women with gestational diabetes mellitus: an integrative review. BMC Pregnancy Childbirth.

[75] McGillivray M, Clarke M (2006). United Nations University Press. Human well-being: Concepts and measures. Understanding human well-being.

[76] Wills-Herrera E, Islam G, Hamilton M (2009). Subjective well-being in cities: A multidimensional concept of individual, social and cultural variables. Appl Res Qual Life.

[77] Diener E, Emmons RA, Larsen RJ, Griffin S (1985). The satisfaction with life scale. J Personal Assess.

[78] Swami V, Weis L, Barron D, Furnham A (2018). Positive body image is positively associated with hedonic (emotional) and eudaimonic (psychological and social) well-being in British adults. J Soc Psychol.

[79] Yildirim M (2019). Mediating Role of Resilience in the Relationships Between Fear of Happiness and Affect Balance, Satisfaction With Life, and Flourishing. Eur J Psychol.

[80] Sexton JB, Adair KC. Forty-five good things: a prospective pilot study of the Three Good Things well-being intervention in the USA for healthcare worker emotional exhaustion, depression, work–life balance and happiness. BMJ Open. 2019. 10.1136/bmjopen-2018-022695. 10.1136/bmjopen-2018-022695 PMC6475256 30898795

[81] Eid M, Larsen RJ (2008). The science of subjective well-being.

[82] Ryan RM, Deci EL (2001). On happiness and human potentials: A review of research on hedonic and eudaimonic well-being. Annu Rev Psychol.

[83] Shoorab NJ (2019). Women’s Experiences of Emotional Recovery from Childbirth-Related Perineal Trauma: A Qualitative Content analysis. Int J Commun Based Nurs Midwifery.

[84] Rasmussen B, Dunning T, Hendrieckx C, Botti M, Speight J (2013). Transition to motherhood in type 1 diabetes: design of the pregnancy and postnatal well-being in transition questionnaires. BMC Pregnancy Childbirth..

[85] Rasmussen B, Hendrieckx C, Clarke B, Botti M, Dunning T, Jenkins A (2013). Psychosocial issues of women with type 1 diabetes transitioning to motherhood: a structured literature review. BMC Pregnancy Childbirth..

[86] Mirzakhani K, Hejazinia Z, Golmakani N, Sardar MA, Shakeri MT (2015). The effect of birth ball exercises during pregnancy on mode of delivery in primiparous women. J Midwifery Reprod Health..

[87] Nosrati A, Mirzakhani K, Golmakani N, Asghari Nekah M, Esmaeili H (2017). Effect of paternal-fetal attachment on maternal-mental health: A randomized clinical trial. J Mazandaran Univ Med Sci.

[88] Nosrati A, Mirzakhani K, Golmakani N, Asghari Nekah SM, Esmaeili H (2018). The Effect of Paternal-Fetal Attachment Training on Marital Satisfaction during Pregnancy. J Midwifery Reprod Health.

[89] Zalewska AM, Nezlek J, Zieba M (2018). Integrated approach to personality and well-being. Polish Psychol Bulletin.

[90] Maddux JE, Trusz S, Babel P (2016). Self-efficacy. Interpersonal and intrapersonal expectancies.

[91] Larson LM, Seipel MT, Shelley MC, Gahn SW, Ko SY, Schenkenfelder M (2019). The academic environment and faculty well-being: The role of psychological needs. J Career Assess.

[92] Mathew NE, Burton KL, Schierbeek A, Črnčec R, Walter A, Eapen V (2019). Parenting preschoolers with autism: Socioeconomic influences on wellbeing and sense of competence. World J Psychiatry.

[93] Janighorban M, Heidari Z, Dadkhah A, Mohammadi F (2018). Women’s needs on bed rest during high-risk pregnancy and postpartum period: a qualitative study. J Midwifery Reprod Health.

[94] Mirzakhani K, Kadivzadeh T, Faridhosseini F, Ebadi A. Pregnant women’s experiences of the conditions affecting marital well-being in high-risk pregnancy: A qualitative study. Int J Commun Based Nurs Midwifery. 2020; In press. 10.30476/ijcbnm.2020.85666.1285 PMC7648861 33178857

[95] McMahon S, Fleury J, editors. Wellness in older adults: A concept analysis. Nurs Forum. 2012;47(1):39–51. 10.1111/j.1744-6198.2011.00254.x PMC3326391 22309381

[96] Ellison CW (1983). Spiritual well-being: conceptualization and measurement. J Psychol Theol.

[97] Cheng Q, Liu X, Li X, Wang Y, Mao T, Chen Y (2019). Improving spiritual well-being among cancer patients: implications for clinical care. Support Care Cancer.

[98] Amorim TV, Souza ÍEO, Moura M, Vasconcelos A, ABA Q, Salimena AMO (2017). Nursing care perspectives in high-risk pregnancy: integrative review. Enfermería Glob.

[99] van Dierendonck D, Díaz D, Rodríguez-Carvajal R, Blanco A, Moreno-Jiménez B (2008). Ryff’s six-factor model of psychological well-being, a Spanish exploration. Soc Indicators Res.

[100] Ryff CD (1989). Happiness is everything, or is it? Explorations on the meaning of psychological well-being. J Personal Soc Psychol.

[101] Ryff CD, Keyes CLM (1995). The structure of psychological well-being revisited. J Pers Soc Psychol.

[102] Kumano M (2018). On the concept of well-being in Japan: feeling shiawase as hedonic well-being and feeling ikigai as eudaimonic well-being. Appl Res Qual Life.

[103] OECD. Publishing, Organisation for Economic Co-operation and Development. OECD guidelines on measuring subjective well-being. https://www.oecd.org/statistics/oecd-guidelines-on-measuring-subjective-well-being-9789264191655-en.htm. Accessed 20 Mar 2013. 24600748

